# Calculation of DC Stark Resonances for the Ammonia Molecule

**DOI:** 10.3390/molecules29071543

**Published:** 2024-03-29

**Authors:** Patrik Pirkola, Marko Horbatsch

**Affiliations:** Department of Physics and Astronomy, York University, Toronto, ON M3J 1P3, Canada; patpirko@my.yorku.ca

**Keywords:** molecular structure, Stark effect, complex scaling method, Breit-Wigner resonances

## Abstract

A model potential previously developed for the ammonia molecule is treated in a single-center partial-wave approximation in analogy with a self-consistent field method developed by Moccia. The latter was used in a number of collision studies. The model potential is used to calculate DC Stark resonance parameters, i.e., resonance positions and shifts using the exterior complex scaling method for the radial coordinate. Three molecular valence orbitals are investigated for fields along the three Cartesian coordinates, i.e., along the molecular axis and in two perpendicular directions. The work extends previous work on the planar-geometry water molecule for which non-monotonic shifts were observed. We find such non-monotonic shifts for fields along the molecular axis. For perpendicular fields, we report the splitting of the 1e orbitals into a fast- and a slow-ionizing orbital.

## 1. Introduction

Research into ionization of ammonia molecules (${\mathrm{NH}}_{3}$) has been an ongoing topic of interest with recently proposed new ideas about multiple ionization (or rather the lack thereof) in the context of experimental fragmentation studies in proton–ammonia collisions at intermediate and high energies [1]. The fragmentation study puts (perhaps) in doubt the validity of an independent electron model approach which was used in various forms to explain total (net) ionization cross sections, as well as doubly differential cross sections which represent the emission properties of ionized electrons, i.e., their distribution over polar angle and energy. Such studies of net ionization differential cross sections were reported in the Born approximation [2] and a continuum distorted wave method [3], which described the earlier measurements [4] quite well. The fact that these experimental data were consistent with net ionization was demonstrated in yet another study by showing the contributions from particular molecular orbitals (MOs) [5]. Most of these studies employed the single-centre Slater-type orbital-based Hartree–Fock calculations of Moccia [6].

From a theoretical perspective, the role of multiple ionization in proton collisions with the ‘isoelectronic’ molecule water ($H_{2}O$) [7,8,9], methane (${\mathrm{CH}}_{4}$) [10], and ammonia [1] was analyzed in the framework of the independent-atom model [11]. An accurate representation of proton–water molecule differential cross sections at an intermediate energy (250 keV) was obtained with a classical trajectory Monte-Carlo method [12] which was based on a three-center model potential. Collision calculations using this model potential have been carried out recently for the ammonia molecule [13]. These works, and the problem with interpreting fragmentation cross sections [1] serve as a motivation to extend our previous studies of Stark resonance parameters for the water molecule within a model potential approach to the case of the ammonia molecule. The main idea of the model potential approach is to avoid the technical difficulties of a self-consistent effective potential.

We are studying the molecule for fixed orientation, i.e., the rotational (and vibrational) degrees of freedom are ignored. In collision problems at intermediate and high energies, this approach is justified by the time scale of the collision process, and orientation averaging is applied when computing probabilities or cross sections, such as, e.g., in Refs. [11,12]. For the DC Stark problem, the fixed orientation with respect to the external field does represent a more serious issue, since over longer time scales the field would act to orient a molecule with permanent dipole moment [14,15]. An exception would be if the molecule was found in a matrix isolation environment, e.g., by trapping in a cold rare gas matrix. This subject has received recently renewed attention in the context of proposals to measure the electron electric dipole moment using diatomic molecules [16,17]. Given that strong electric fields are potentially going to be applied (cf. Ref. [18]), the problem of Stark ionization should be researched in this context, as well.

The Stark resonance problem is addressed in this paper by following the exterior complex scaling (ECS) method which implements a derivative discontinuity at the radial distance where complex scaling sets in [19]. The ECS methodology was developed over the years and has been compared to the complex absorbing potential (CAP) method [20,21]. Following Moiseyev [22,23], one can argue that the smooth ECS and CAP methods are equivalent. They share the features that starting at some critical radial distance $r_{s}$ either a gradual continuation of the real *r*-axis into the complex plane is carried out, or a complex absorber is implemented for $r>r_{s}$. Both methods show some dependence on either the scaling angle $\theta_{s}$ which extends the path into the complex plane, or on the strength parameter of the CAP. Perturbative corrections can be employed in the case of the CAP.

The hard ECS method was developed further by Scrinzi [24] as an effective absorber for time-dependent problems. A derivative discontinuity in the wave function at the scaling radius $r_{s}$ needs to be implemented, and it allows for the choice of scaling angles close to the critical value of $\theta_{s}=\pi /2$. This, in turn, allows one to use a reduced region $r_{s}<r<r_{\max}$ to compute the tails of the resonance states. At the outer boundary $r_{\max}$, the Dirichlet condition of vanishing wave function is applied. We used his methodology previously for the planar water molecule [25]. The extension from a planar geometry does not pose additional problems for the present case of the ammonia molecule: a partial wave expansion of the orbitals is implemented, and as before, the radial functions are solved using a finite element method. The choices for scaling radius $r_{s}=16.2$ a.u. and $r_{\max}=24.3$ a.u. were made in this work combined with a scaling angle $\theta_{s}=0.9\;\pi /2$. Atomic units ($\hbar =m_{e}=e=4\pi \epsilon_{0}=1$) are used throughout this work.

**Figure 1:**
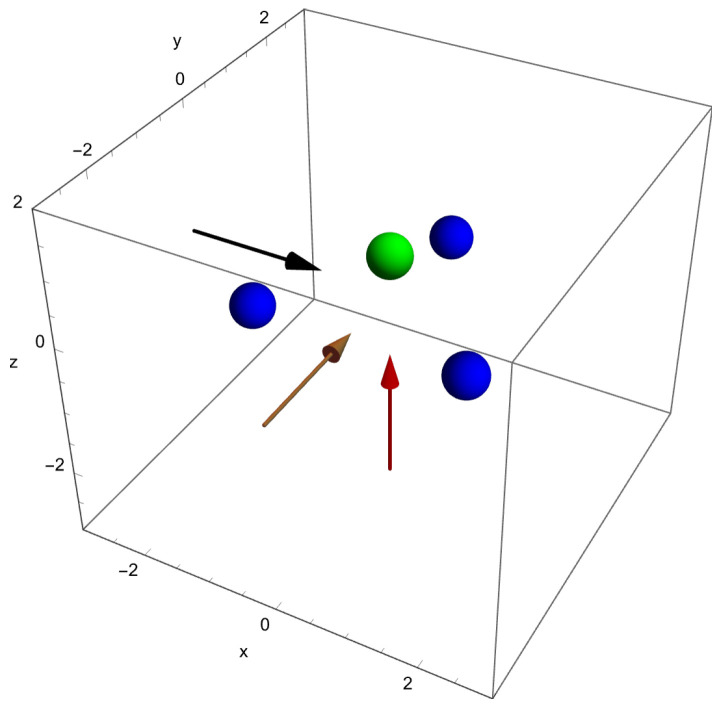
The geometry for the ${\mathrm{NH}}_{3}$ molecule as implemented in this work showing the nitrogen atom (in green) and the three hydrogen atoms (in blue) schematically. Electric fields are applied pushing electrons out along the *x*-direction (black arrow), the *y*-direction (brown arrow), and the *z*-direction (red arrow), and will be denoted by positive values of $F_{x},F_{y},F_{z}$, respectively. Negative values of $F_{x},F_{y},F_{z}$ correspond to fields pushing in the opposite directions. The coordinates are given in atomic units.

The geometry of the ${\mathrm{NH}}_{3}$ molecule is shown in Figure 1 together with the three directions along which electric fields are applied. The arrows indicate the force directions that are applied individually, i.e., one Cartesian direction at a time. The force directions $F_{i}$ are opposite to the electric field directions $E_{i}$ due to the negative charge of the electron.

## 2. Model

The model potential for the ammonia molecule is a straightforward extension of previous modelling of the water molecule (Refs. [26,27,28,29,30]). The model combines three spherically symmetric potentials for the atomic constituents. Each part contains a screening contribution, and the parameters are adjusted such that the overall potential falls as −1/r at large distances, as is required to avoid contributions from electronic self-interaction. We keep the model parameters for the hydrogen atoms, and model the central nitrogen atom in analogy to the oxygen atom in $H_{2}O$.

Thus, the potential is defined as follows:

(1) $$V_{\mathrm{eff}}=V_{N}\left(r\right)+V_{H}\left(r_{1}\right)+V_{H}\left(r_{2}\right)+V_{H}\left(r_{3}\right)\;,$$

(2) $$\begin{array}{c} \begin{array}{cc} V_{N}\left(r\right)= & -\frac{7-N_{N}}{r}-\frac{N_{N}}{r}\left(1+\alpha_{N}r\right)\exp\left(-2\alpha_{N}r\right)\;,\\ V_{H}\left(r_{j}\right)= & -\frac{1-N_{H}}{r_{j}}-\frac{N_{H}}{r_{j}}\left(1+\alpha_{H}r_{j}\right)\exp\left(-2\alpha_{H}r_{j}\right)\;. \end{array} \end{array}$$

The scalar variables $r_{j}$ (with j=1,2,3) represent the electron distances from the protons. The hydrogenic parameters $\alpha_{H}=0.6170$ and $N_{H}=0.9075$ are taken from previous works for the water molecule. The latter choice then fixes the potential parameter $N_{N}=6.2775$ to yield the appropriate asymptotic effective potential at large *r*, as $3\;(1-N_{H})=0.2775$ is the long-range effective charge contribution to the potential from the hydrogen atoms. The nitrogen atom screening parameter was chosen as $\alpha_{N}=1.525$ in order for the model to yield orbital energies that follow closely values obtained in the Hartree–Fock approximation. The geometry of ${\mathrm{NH}}_{3}$ is adopted from the work of Moccia [6], with a N-H bond length of 1.928a.u., polar angle $\theta_{p}=108.9$ degrees, and azimuthal angles $\phi_{j}$ for the three hydrogenic protons spaced apart by 120 degrees. In particular, the azimuthal angles of the hydrogen atoms are chosen to be 90, 210, 330 degrees, which singles out the y-z plane as containing one of the protons.

The Schrödinger equation for the MOs and an electric field in the $\hat{z}$ direction can be written as

(3) $$\left[-\frac{1}{2}\nabla^{2}-\sum_{i=0}^{3}\frac{Z_{i}\left(\right|{\vec{r}}_{i}\left|\right)}{|{\vec{r}}_{i}|}-F_{z}r\cos\left(\theta \right)\right]\psi_{\nu }=\epsilon_{\nu }\;\psi_{\nu },$$

with $r_{0}\equiv r$, while the $Z_{i}\left(r_{i}\right)$ are screening functions for the constituent atomic centers, i.e., $Z_{0}\left(r\right)=rV_{N}\left(r\right)$ for the nitrogen atom, and $Z_{i}\left(r\right)=rV_{H}\left(r\right)$ with i=1,2,3 for the hydrogens, as defined in Equation (2). Note that the electric field component is $E_{z}=-F_{z}$, i.e., our notation $F_{z}$ refers to the force experienced by a free electron.

The MO wavefunctions $\psi \equiv \psi_{\nu }$ are expanded in complex-valued spherical harmonics,

(4) $$\psi \left(r,\theta ,\phi \right)=\sum_{\ell =0}^{\ell_{\max}}\sum_{m=-\ell }^{\ell }\sum_{i,n}^{I,N}c_{in\ell m}\frac{f_{in}\left(r\right)}{r}Y_{\ell }^{m}\left(\theta ,\phi \right)\;,$$

and the radial functions are expanded using a finite-element method (FEM). The functions $f_{in}$ are local basis functions on interval *i* of the radial interval $0<r<r_{\max}$. The index *n* labels the polynomial basis functions [19]. The Schrödinger equation is solved as outlined in Refs. [25,31] and leads to a matrix eigenvalue problem with the $c_{in\ell m}$ being elements of the eigenvectors. With this discretization technique we are solving the three-dimensional problem, which is defined in Equation (3) for a field along the $\hat{z}$ direction. The force direction due to the external DC field as experienced by the electron is controlled by the sign of $F_{z}$ as explained in Figure 1.

The FEM approach from Refs. [25,31] and outlined in Refs. [19,32] was used with the partial wave expansion of ψ truncated at $\ell_{\max}=3$ to test how the MO eigenvalues respond to changes in the one free screening parameter contained in the model potential. The partial wave expansion allowed the spherical components of the matrix elements to be calculated using a Wigner 3j coefficient package [33] (which can be found on the author(s) homepage (https://www-stone.ch.cam.ac.uk/wigner.shtml accessed on 28 March 2024)) as before in our work with water [25,31,32]. The hydrogenic potentials are expanded in spherical harmonics, which allows for the use of Wigner 3j coefficients rather than evaluating three-dimensional integrals numerically.

Comparison with the SCF eigenvalues of Moccia shows that the three outermost MOs can be reproduced well with the simple model potential. The $2a_{1}$ MO is too weakly bound at the level of 10%, which is deemed acceptable, since it is expected to contribute less to the overall molecular ionization rate. The comparison of eigenvalues obtained for $\ell_{\max}=3$ and $\ell_{\max}=5$ is provided, since the resonance parameter calculations are performed with $\ell_{\max}=3$ only. Table 1 also contains results from a localized Hartree–Fock method as implemented in Turbomole [34,35,36,37].

## 3. Results

### 3.1. Resonance Parameters for Fields along the Vertical $\pm \hat{z}$-Direction

We begin the discussion of resonance parameters with fields along $\pm \hat{z}$ for which the two degenerate MOs $1e_{1}$ and $1e_{2}$ should yield identical results. The dominant contribution to DC field ionization is expected from the weakest bound orbital ($3a_{1}$). For this orbital the combined molecular and external electric field leads to over-the-barrier ionization at the strongest fields calculated ($|F_{z}|$ of order 0.1a.u.).

**Figure 2:**
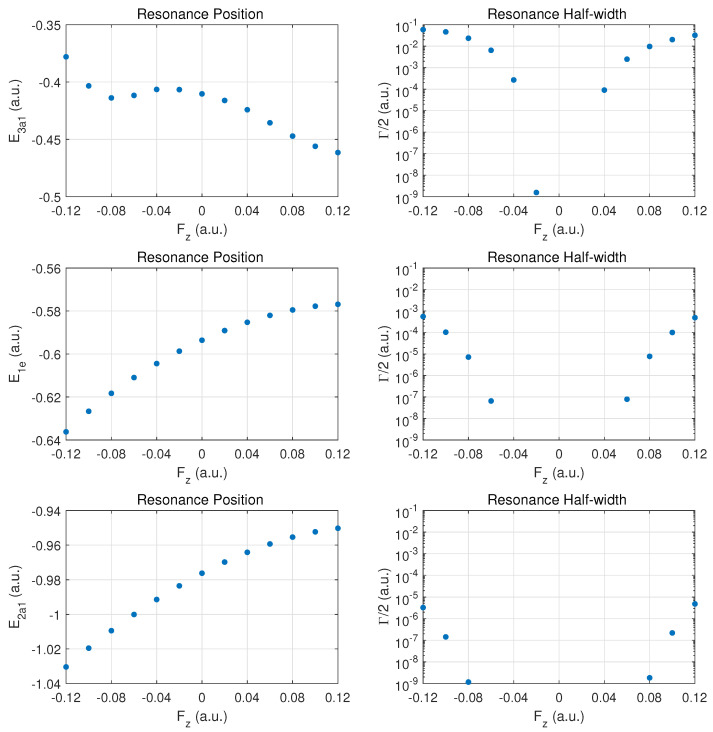
Resonance positions (**left panels**) and half widths (**right panels**) in atomic units for the outer MOs for electric fields along the axis connecting the nitrogen atom with the atomic hydrogen plane. $F_{z}>0$ values correspond to fields pushing electrons out on the nitrogen side, while $F_{z}<0$ corresponds to emission from the side of the hydrogen atom plane. Top row: $3a_{1}$; middle row: the doubly degenerate 1e MO (identical results for $1e_{1}$ and $1e_{2}$), bottom row for $2a_{1}$.

In Figure 2 the top row shows the resonance position (left column) and resonance half width (right column) for this orbital as a function of field strength $F_{z}$. Positive values $F_{z}>0$ correspond to the field direction pushing electrons out in the direction from the hydrogen atom plane past the nitrogen atom, while negative values $F_{z}<0$ are for electrons ejected from the hydrogen plane (at negative *z*) away from the nitrogen atom which is located at z=0.

The change of the resonance position with field strength can be described as monotonically stronger binding for $F_{z}>0$, since electron density is transferred from the hydrogen atoms in the direction of the central nitrogen atom. For the opposite field direction ($F_{z}<0$), we observe non-monotonic behavior. First, one expects marginally weaker binding when transferring electron density from a nitrogen to a hydrogen atom.

In the molecule, the shift in electron density will be towards regions around the partially shielded protons where the electron binding is weaker. This feature becomes apparent at strong fields (over-the-barrier regime), but there is an intermediate range of field strengths ($-0.08<F_{z}<-0.04\;a.u.$) where there is a non-monotonic variation in the resonance position with field strength.

The resonance widths are obviously small in the tunneling regime. They change by orders of magnitude as the field is increased, and the ionization rate for emission from the hydrogen plane ($F_{z}<0$) is stronger than in the opposite direction, by more than a factor of two. For field strengths of the order of 0.1a.u., saturation in the ionization rate sets in, which is associated with the over-the-barrier regime. At these field strengths, one may reach the limitations of the exponential decay model, and, thus, results for stronger fields are not reported.

We note that the behavior of the resonance position is consistent with the change in ionization rate (or resonance width) as a function of field direction. In the strong field regime (at about 0.1a.u. and beyond), the binding energies are quite different and the ionization rates change by a factor of two when the field is reversed. An interesting observation for $F_{z}>0$ is the rise in the ionization rate even though the resonance position indicates stronger binding. This phenomenon is associated with density being driven by the field towards the barrier region.

In the middle panel, the results are shown for the two degenerate 1e MOs. The dependence of the resonance position on field strength $F_{z}$ is monotonic in this case, and varies only at the 5% level in the given field strength range. The corresponding decay rates are weaker by orders of magnitude as compared to the $3a_{1}$ MO, and remain in the tunneling regime. This conclusion will be supported further below by probability density plots for $|F_{z}|=0.1\;a.u.$

The bottom panel shows results for the more deeply bound $2a_{1}$ MO. Here, the variation in the resonance position is only at the level of 3%, and the ionization rate is suppressed by two to three additional orders of magnitude. The shape of the DC Stark shift (left panel) as a function of field strength and orientation is similar to what is observed for the 1e pair of MOs. A small asymmetry can be observed in the decay rates, with a small enhancement for $F_{z}>0$ vs. $F_{z}<0$.

**Figure 3:**
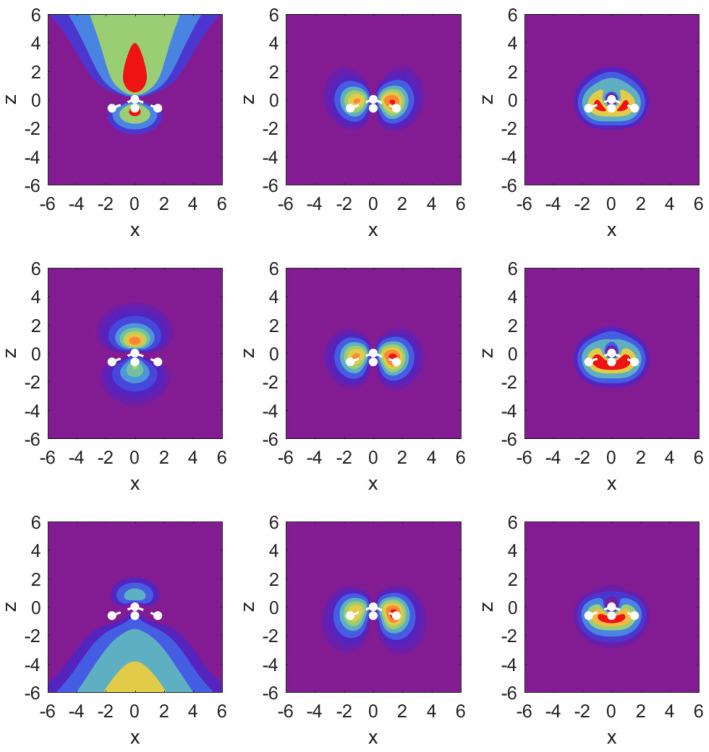
Probability density contour plots for the MOs $3a_{1}$, $1e_{1}$, $2a_{1}$ (left to right) in the y=0 plane, i.e., as a function of *x* and *z*. Middle row: field-free case; top row: $F_{z}=0.1\;a.u.$; bottom row: $F_{z}=-0.1\;a.u.$ The contours are at values: [0.00,0.005,0.01,0.02,0.04,0.06,0.08,0.1,0.12,0.14]. The positions of the atomic nuclei are indicated by white dots with the N atom at z=0 and the three proton locations projected onto the x-z plane. The central dot corresponds to the proton residing below the positive *y*-axis.

In Figure 3, we illustrate the situation with probability density contour plots of the MOs. The field-free case is shown in the middle row. The outermost MO ($3a_{1}$) shown on the left has an asymmetric probability density with respect to z=0 with higher probability values on the nitrogen side. When the DC field is pushing electrons out on this side, the nitrogen potential provides attraction and causes some concentration of probability in this distribution, as shown in the top left panel (strong red drop-like shape at z>0 and also at z<0). The interpretation of the density plots is that they describe steady-state decay.

The bottom left panel shows the case of a strong field pushing in the direction past the hydrogen atoms. The probability distribution is more diffuse, showing that the outflow on the side of the hydrogen plane is hindered less. This observation is consistent with the decay rate results shown in the top right panel of Figure 2. The outflow of probability density is consistent with above-the-barrier ionization for both the top and bottom rows, i.e., $|F_{z}|=0.1\;a.u.$

The other two MOs show much less outflow at comparable fields, and are clearly in the tunneling regime. For the $1e_{1}$ MO (middle column) with field turned on in either direction, there is a limited amount of density change compared to the $3a_{1}$ MO. For the $2a_{1}$ MO (right column), we observe symmetry in the field-free case, and shifting of probability density in the direction of the applied force, but in the tunneling regime not much probability density appears far away from the molecule.

### 3.2. Resonance Parameters for Fields along the $\pm \hat{x}$-Direction

**Figure 4:**
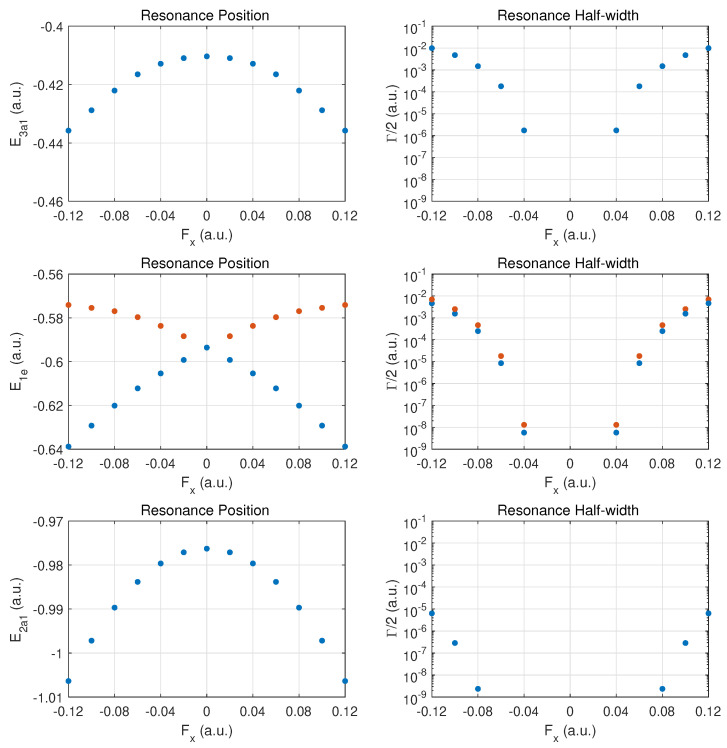
Same as in Figure 2 but for electric DC fields along the *x*-axis. In the middle panel, results are shown for the MOs $1e_{1}$ (orange dots) and $1e_{2}$ (blue dots). The $1e_{1}$ MO has the larger ionization rate for this field orientation.

In Figure 4, results are presented for fields in a perpendicular direction relative to the axis connecting the N atom with the hydrogen atom plane. The arrangement of the three hydrogen atoms is such that one resides on the *y*-axis; i.e., field emission occurs along the direction of the H-H bond perpendicular to this axis. The degeneracy between the $1e_{1}$ and $1e_{2}$ MO energies is expected to be broken when a DC field is applied in this $\hat{x}$ (or the perpendicular $\hat{y}$) direction.

The top row for the outermost MO $3a_{1}$ shows a symmetric behaviour in the DC Stark shift (left panel) and likewise a symmetric ionization rate with respect to reversal of the field direction. The change in the rise of the ionization rate at strong fields indicates that one is only approaching the over-the-barrier regime, i.e., saturation has not set in yet at $|F_{x}|=0.1\;a.u.$ The increase in binding is at the 10% level for the strongest fields. The ionization rates for these fields are smaller than for ionization along the $\hat{z}$ axis by a substantial factor (about three or six, depending on the field direction $\pm \hat{z}$).

The middle row shows the different behaviors for the $1e_{1}$ and $1e_{2}$ MOs. We classify the two orbitals as fast- vs. slow-ionizing under $\hat{x}$ oriented fields (orange vs. blue markers). The behavior is symmetric with respect to field orientation. The $1e_{1}$ MO (orange dots) shifts towards less binding for both field orientations, and the $1e_{2}$ (blue dots) is bound more deeply as the field is increased in either direction.

The ionization rates (right panel) are also symmetrical with respect to field reversal. The $1e_{1}$ ionizes more readily by almost a factor of two for this field orientation. It is remarkable that these MOs ionize easily at strong fields with the $1e_{1}$ MO displaying a rate which is moving towards that of the more weakly bound $3a_{1}$ MO. Comparing the ionization rates for the $1e_{1}$ and $1e_{2}$ orbitals for fields along $\hat{x}$ versus $\hat{z}$, we notice an order-of-magnitude increase. The reverse trend is true for the $3a_{1}$ MO.

The bottom row shows that the results for the $2a_{1}$ MO are symmetric with respect to field orientation (as for 3a1). The decay rates are somewhat larger than in the case of *z*-oriented fields, even though the MO is bound more deeply with increasing field strength.

**Figure 5:**
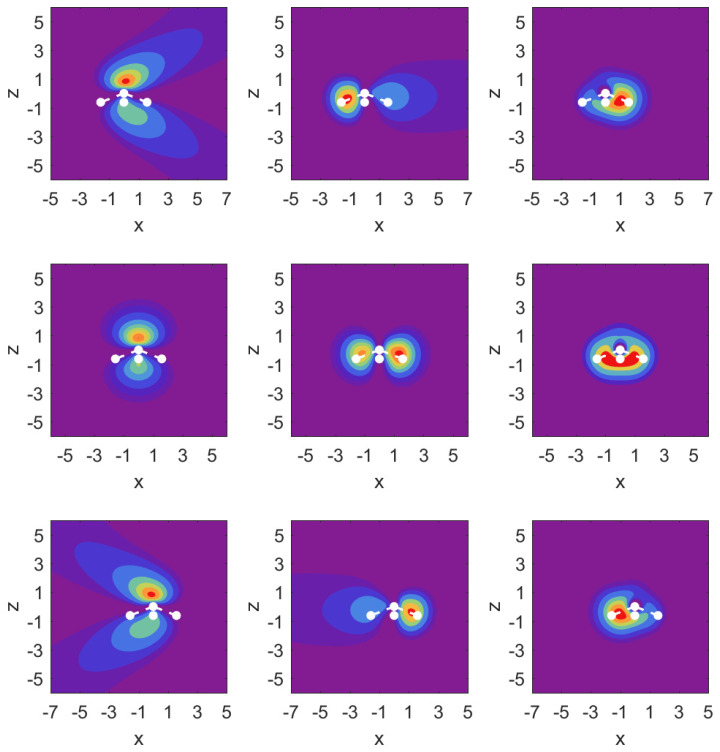
Same as in Figure 3, but for DC electric fields along the $\hat{x}$ axis. Left to right: MOs $3a_{1}$, $1e_{1}$, $2a_{1}$; middle panel: field-free, top panel $F_{x}=+0.1\;a.u.$, bottom panel $F_{x}=-0.1\;a.u.$

We support our resonance parameter values again with selective plots of probability densities from the ECS approach in Figure 5. The middle row is identical with that in Figure 5, but is included for direct comparison of the cases with electric field. It is immediately apparent that three MOs ($3a_{1}$, $1e_{1}$, and also $1e_{2}$, which is not shown) contribute strongly to ionization of the molecule for this field orientation.

For the $3a_{1}$ MO, we observe outflow in the form of two jets directed above and below the hydrogen atom plane. For the $1e_{1}$ MO, we find that the apparent asymmetry in the *x*-*z* plane for the field-free case has no repercussions for the outflow in the case with fields of either direction: both cases show very symmetric probabilities under these conditions, which is consistent with the findings for the resonance parameters.

For the $2a_{1}$ MO shown in the right column, symmetry with respect to field orientation is expected. This is evident from the density plots by comparing the top and bottom panels. We note the relatively strong effect the field has on this relatively deeply bound orbital.

### 3.3. Resonance Parameters for Fields along the $\pm \hat{y}$-Direction

**Figure 6:**
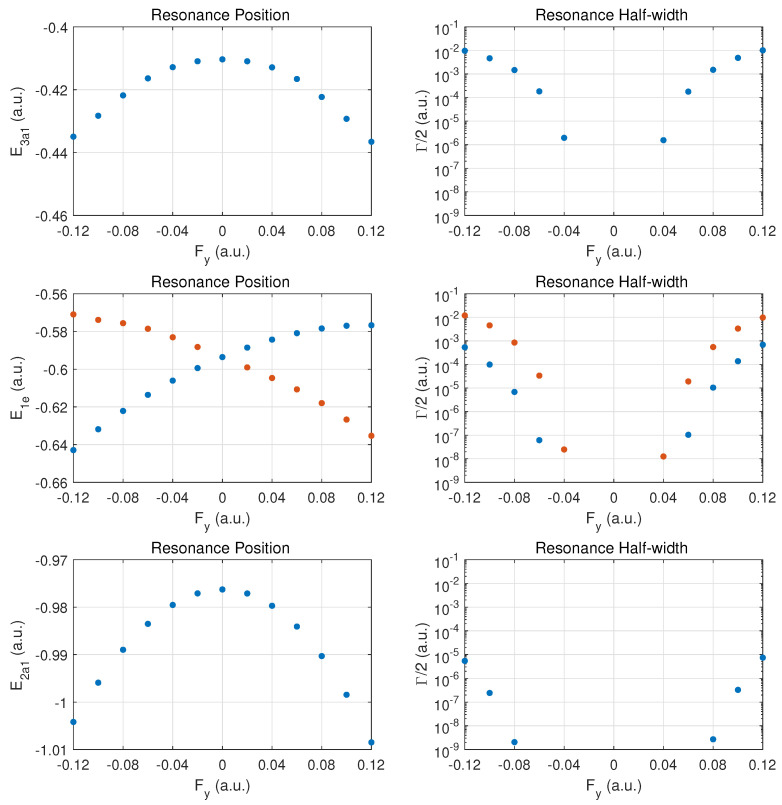
Same as in Figure 4 but for electric DC fields along the *y*-axis. In the middle panel, results are shown for the MOs $1e_{1}$ (blue dots) and $1e_{2}$ (orange dots), which are classified as the slow- vs. fast-ionizing 1e orbital, respectively. Note that the *y*-*z* plane contains a hydrogen atom at y>0 and this causes an asymmetry in the resonance parameters with respect to the sign of $F_{y}$.

In Figure 6, results are given for field orientation along $\hat{y}$. Given the triangular nature of the hydrogen atom plane, these results differ strongly from those in the previous section. By choice of azimuthal angles $\phi_{i}=90,210,330$ degrees, a proton is located on the positive *y* axis, and asymmetry is expected when reversing the field direction.

The top row shows that this asymmetry plays a very small role for the outermost MO $3a_{1}$ at weak fields, and is barely noticeable. The tabulated data (cf. Appendix A.3) show that the shifts differ by less than a percent.

The $1e_{1}$ and $1e_{2}$ MOs (blue and orange dots, middle row) show markedly different behavior when compared to $\pm \hat{x}$ oriented fields. The shifts follow monotonic curves, as there is no longer symmetry under field orientation reversal. The $1e_{2}$ MO is ionizing more rapidly as compared to $1e_{1}$ by about two orders of magnitude. The variation in resonance position is clearly at odds with this result; i.e., it apparently does not play a role here. As discussed before, the actual value of the MO binding energy is not the deciding factor, but rather how the electron density is driven towards the potential barrier by the external field.

The $1e_{1}$ MO ionizes very weakly, and its rate is comparable to those obtained for $\pm \hat{z}$ oriented fields. Thus, one may conclude that the $1e_{2}$ MO is affected by this field orientation dramatically.

In the third row, we give results for the deeply bound $2a_{1}$ MO which remains deeply in the tunneling regime for the given field strengths. It shows a small amount of asymmetry in the DC Stark shifts and in the decay rates.

**Figure 7:**
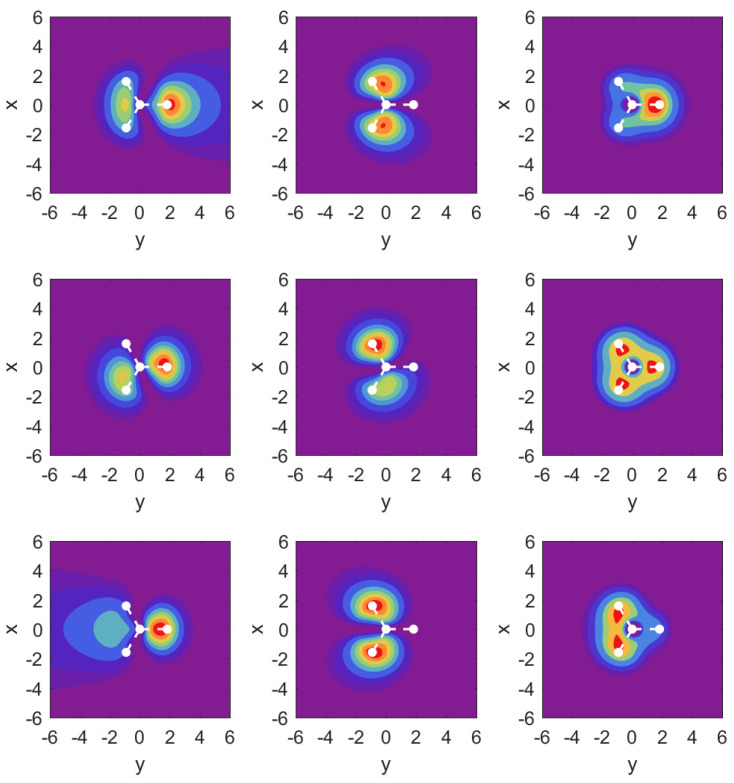
Probability densities in the z=0 plane for DC fields along the $\hat{y}$ direction. Left column: MO $1e_{2}$; middle column: $1e_{1}$; right column: $2a_{1}$ are shown over the *x*-*y* plane. Middle row shows the field-free case; top row: $F_{y}=0.1\;a.u.$; bottom row: $F_{y}=-0.1\;a.u.$

The probability density plots shown in Figure 7 again help to understand the finding for the parameter values. The results for MO $3a_{1}$ are very close to the corresponding plots in Figure 5 and are not shown. Instead, we show over the x-y plane probability densities for the strongly ionizing $1e_{2}$ MO (left column), the much more weakly ionizing $1e_{1}$ MO (middle column), and $2a_{1}$ in the right column.

For the field-free case, the density plots show that the MOs $1e_{2}$ and $1e_{1}$ are actually not perfectly aligned with our *x*- and *y*-axes. This is related to the fact that they share the same probability density shapes, except that they are rotated by 90 degrees, and this is incompatible with the three-fold symmetry. Once the strong field is turned on along either the *x*- or the *y*-axis, however, the densities respect the symmetry of the external field, and one can identify $1e_{1}=1e_{x}$ and $1e_{2}=1e_{y}$.

The results for the $1e_{2}$ MO (left column) show an asymmetry in the outflow for the case $F_{y}>0$ vs. $F_{y}<0$. It is interesting to observe that while the shape of the outflow is very different for both cases, the ionization rates actually differ only at the level of up to 50% (cf. Appendix A.3).

In the middle column for MO $1e_{1}$, we observe a symmetry in the outflows despite the fact that the arrangement of hydrogen atoms is asymmetric. For the case of MO $2a_{1}$, we find that the central parts of the density are very different for the two field directions, but the parts showing the outflow are again very similar, and this is similar to what one observes for MO $1e_{1}$.

## 4. Conclusions

We have extended our previous model calculation for $H_{2}O$ to the case of ${\mathrm{NH}}_{3}$. A simple model potential was applied to approximate what an SCF model (e.g., local density functional theory) might obtain for DC field ionization. In fact, the MO eigenvalues were shown to be comparable to the LHF results, as shown in Table 1.

The results demonstrate that depending on the orientation of the electric field the three outer MOs of ammonia, i.e., $3a_{1}$, $1e_{1}$, and $1e_{2}$ can have appreciable ionization rates. This supports the case for multiple ionization being important whether in ion–molecule collisions or in strong-field laser–molecule interactions.

Interesting details emerge from our model calculations: *(i)* non-monotonic DC shifts for the 3a1 MO for fields along $\hat{z}$; *(ii)* for fields perpendicular to the molecular axis, we observe that the 1e orbitals separate into fast- and slow-ionizing ones; the *(iii)* fast-ionizing 1e orbital can acquire an ionization rate comparable to the outermost 3a1 orbital.

It would be of interest to test these predictions with more sophisticated models, such as Hartree–Fock theory for MO ionization rates, density functional theory (DFT) with electron correlation, or even coupled-cluster theory of net ionization, which is possible within a recently developed quantum chemistry code [38,39,40]. Work is in progress to replace the model potential by exchange-correlation potentials obtained from DFT. 

## Figures and Tables

**Table 1:** MO eigenvalues for the model potential as compared to the SCF eigenvalues of Moccia (Ref. [6]). The $E_{1e}$ energy appears twice, i.e., for the MOs $1e_{1}$ and $1e_{2}$. The fourth row shows the localized HF method [34,35] eigenvalues based on the optimized geometry in HF approximations as calculated in Turbomole [37] using the def2-QZVPPD basis set, while the fifth row gives the eigenvalues from the optimized effective potential method [36] using the d-aug-cc-pVTZ-oep basis.

	$E_{1a1}$	$E_{2a1}\;$	$E_{1e}$	$E_{3a1}$
Ref. [6]	−15.5222	−1.1224	−0.5956	−0.4146
FEM ($\ell_{\max}=3$)	−15.930	−0.976	−0.594	−0.410
FEM ($\ell_{\max}=5$)	−15.930	−0.982	−0.609	−0.413
LHF	−14.087	−0.986	−0.619	−0.424
OEP-EXX	−14.154	−0.986	−0.611	−0.430

## Data Availability

Data are contained within the article.

## Appendix A. Tables of Resonance Parameter Values

### Appendix A.1. Fields along ±$\hat{z}$

MO: $3a_{1}$		
$F_{z}$	Re	Im
−0.12	−0.3780	−5.893 × 10^−2^
−0.10	−0.4034	−4.630 × 10^−2^
−0.08	−0.4139	−2.349 × 10^−2^
−0.06	−0.4117	−6.434 × 10^−3^
−0.04	−0.4065	−2.686 × 10^−4^
−0.02	−0.4066	−1.556 × 10^−9^
0.00	−0.4103	NA
0.02	−0.4161	NA
0.04	−0.4242	−9.049 × 10^−5^
0.06	−0.4356	−2.484 × 10^−3^
0.08	−0.4472	−9.694 × 10^−3^
0.10	−0.4561	−2.027 × 10^−2^
0.12	−0.4615	−3.230 × 10^−2^

MO: 1e		
$F_{z}$	Re	Im
−0.12	−0.6362	−5.524 × 10^−4^
−0.10	−0.6267	−1.032 × 10^−4^
−0.08	−0.6183	−7.178 × 10^−6^
−0.06	−0.611	−6.516 × 10^−8^
−0.04	−0.6045	NA
−0.02	−0.5987	NA
0.00	−0.5936	NA
0.02	−0.5891	NA
0.04	−0.5853	NA
0.06	−0.5820	−7.860 × 10^−8^
0.08	−0.5795	−7.737 × 10^−6^
0.10	−0.5778	−1.003 × 10^−4^
0.12	−0.5769	−4.911 × 10^−4^

MO: $2a_{1}$		
$F_{z}$	Re	Im
−0.12	−1.030	−3.287 × 10^−6^
−0.10	−1.020	−1.437 × 10^−7^
−0.08	−1.009	−1.161 × 10^−9^
−0.06	−1.000	NA
−0.04	−0.9914	NA
−0.02	−0.9835	NA
0.00	−0.9763	NA
0.02	−0.9698	NA
0.04	−0.9642	NA
0.06	−0.9594	NA
0.08	−0.9554	−1.834 × 10^−9^
0.10	−0.9523	−2.197 × 10^−7^
0.12	−0.9503	−4.861 × 10^−6^

### Appendix A.2. Fields along ±$\hat{x}$

MO: $3a_{1}$		
$F_{x}$	Re	Im
−0.12	−0.4358	−9.906 × 10^−3^
−0.10	−0.4288	−4.740 × 10^−3^
−0.08	−0.4221	−1.488 × 10^−3^
−0.06	−0.4165	−1.803 × 10^−4^
−0.04	−0.4129	−1.736 × 10^−6^
−0.02	−0.4109	NA
0	−0.4103	NA
0.02	−0.4109	NA
0.04	−0.4129	−1.736 × 10^−6^
0.06	−0.4165	−1.803 × 10^−4^
0.08	−0.4221	−1.488 × 10^−3^
0.10	−0.4288	−4.740 × 10^−3^
0.12	−0.4358	−9.906 × 10^−3^

MO: $1e_{1}$ (fast)		
$F_{x}$	Re	Im
−0.12	−0.5741	−6.925 × 10^−3^
−0.10	−0.5754	−2.515 × 10^−3^
−0.08	−0.5770	−4.623 × 10^−4^
−0.06	−0.5797	−1.799 × 10^−5^
−0.04	−0.5837	−1.296 × 10^−8^
−0.02	−0.5884	NA
0	−0.5936	NA
0.02	−0.5884	NA
0.04	−0.5837	−1.296 × 10^−8^
0.06	−0.5797	−1.799 × 10^−5^
0.08	−0.5770	−4.623 × 10^−4^
0.10	−0.5754	−2.515 × 10^−3^
0.12	−0.5741	−6.925 × 10^−3^

MO: $1e_{2}$ (slow)		
$F_{x}$	Re	Im
−0.12	−0.6388	−4.592 × 10^−3^
−0.10	−0.6292	−1.551 × 10^−3^
−0.08	−0.6201	−2.475 × 10^−4^
−0.06	−0.6122	−8.496 × 10^−6^
−0.04	−0.6054	−5.725 × 10^−9^
−0.02	−0.5992	NA
0	−0.5936	NA
0.02	−0.5992	NA
0.04	−0.6054	−5.730 × 10^−9^
0.06	−0.6122	−8.496 × 10^−6^
0.08	−0.6201	−2.475 × 10^−4^
0.10	−0.6292	−1.551 × 10^−3^
0.12	−0.6388	−4.592 × 10^−3^

MO: $2a_{1}$		
$F_{x}$	Re	Im
−0.12	−1.006	−6.357 × 10^−6^
−0.10	−0.9972	−2.855 × 10^−7^
−0.08	−0.9897	−2.367 × 10^−9^
−0.06	−0.9838	NA
−0.04	−0.9796	NA
−0.02	−0.9771	NA
0	−0.9763	NA
0.02	−0.9771	NA
0.04	−0.9796	NA
0.06	−0.9838	NA
0.08	−0.9897	−2.366 × 10^−9^
0.10	−0.9972	−2.855 × 10^−7^
0.12	−1.006	−6.357 × 10^−6^

### Appendix A.3. Fields along ±$\hat{y}$

MO: $3a_{1}$		
$F_{y}$	Re	Im
−0.12	−0.4349	−9.669 × 10^−3^
−0.10	−0.4283	−4.644 × 10^−3^
−0.08	−0.4218	−1.471 × 10^−3^
−0.06	−0.4164	−1.833 × 10^−4^
−0.04	−0.4128	−1.962 × 10^−6^
−0.02	−0.4109	NA
0	−0.4103	NA
0.02	0.411	NA
0.04	−0.4129	−1.559 × 10^−6^
0.06	−0.4166	−1.788 × 10^−4^
0.08	−0.4223	−1.506 × 10^−3^
0.10	−0.4293	−4.825 × 10^−3^
0.12	−0.4366	−1.011 × 10^−2^

MO: $1e_{2}$ (fast)		
$F_{y}$	Re	Im
−0.12	−0.571	−1.201 × 10^−2^
−0.10	−0.5739	−4.555 × 10^−3^
−0.08	−0.5756	−8.496 × 10^−4^
−0.06	−0.5786	−3.357 × 10^−5^
−0.04	−0.5831	−2.476 × 10^−8^
−0.02	−0.5882	NA
0	−0.5936	NA
0.02	−0.5991	NA
0.04	−0.6047	−1.254 × 10^−8^
0.06	−0.6107	−1.906 × 10^−5^
0.08	−0.6180	−5.480 × 10^−4^
0.10	−0.6267	−3.325 × 10^−3^
0.12	−0.6353	−9.772 × 10^−3^

MO: $1e_{1}$ (slow)		
$F_{y}$	Re	Im
−0.12	−0.6429	−5.352 × 10^−4^
−0.10	−0.6318	−9.919 × 10^−5^
−0.08	−0.6221	−6.839 × 10^−6^
−0.06	−0.6136	−6.150 × 10^−8^
−0.04	−0.6061	NA
−0.02	−0.5994	NA
0	−0.5936	NA
0.02	−0.5886	NA
0.04	−0.5843	NA
0.06	−0.5809	−1.040 × 10^−7^
0.08	−0.5784	−1.043 × 10^−5^
0.10	−0.5770	−1.380 × 10^−4^
0.12	−0.5767	−6.887 × 10^−4^

MO: $2a_{1}$		
$F_{y}$	Re	Im
−0.12	−1.004	−5.367 × 10^−6^
−0.10	−0.9959	−2.436 × 10^−7^
−0.08	−0.9890	−2.045 × 10^−9^
−0.06	−0.9835	NA
−0.04	−0.9795	NA
−0.02	−0.9771	NA
0	−0.9763	NA
0.02	−0.9771	NA
0.04	−0.9797	NA
0.06	−0.9841	NA
0.08	−0.9903	−2.701 × 10^−9^
0.10	−0.9984	−3.287 × 10^−7^
0.12	−1.008	−7.362 × 10^−6^
